# ARA-PEPs: a repository of putative sORF-encoded peptides in *Arabidopsis thaliana*

**DOI:** 10.1186/s12859-016-1458-y

**Published:** 2017-01-17

**Authors:** Rashmi R. Hazarika, Barbara De Coninck, Lidia R. Yamamoto, Laura R. Martin, Bruno P. A. Cammue, Vera van Noort

**Affiliations:** 1 KU Leuven, Centre of Microbial and Plant Genetics, Kasteelpark Arenberg 20, Leuven, B-3001 Belgium; 2 Department of Plant Systems Biology, VIB, Technologiepark 927, Ghent, B-9052 Belgium

**Keywords:** RNA-seq, Tiling arrays, sORFs, Database, Peptides, *Arabidopsis thaliana*

## Abstract

**Background:**

Many eukaryotic RNAs have been considered non-coding as they only contain short open reading frames (sORFs). However, there is increasing evidence for the translation of these sORFs into bioactive peptides with potent signaling, antimicrobial, developmental, antioxidant roles etc. Yet only a few peptides encoded by sORFs are annotated in the model organism *Arabidopsis thaliana*.

**Results:**

To aid the functional annotation of these peptides, we have developed ARA-PEPs (available at http://www.biw.kuleuven.be/CSB/ARA-PEPs), a repository of putative peptides encoded by sORFs in the *A. thaliana* genome starting from in-house Tiling arrays, RNA-seq data and other publicly available datasets. ARA-PEPs currently lists 13,748 sORF-encoded peptides with transcriptional evidence. In addition to existing data, we have identified 100 novel transcriptionally active regions (TARs) that might encode 341 novel stress-induced peptides (SIPs). To aid in identification of bioactivity, we add functional annotation and sequence conservation to predicted peptides.

**Conclusion:**

To our knowledge, this is the largest repository of plant peptides encoded by sORFs with transcript evidence, publicly available and this resource will help scientists to effortlessly navigate the list of experimentally studied peptides, the experimental and computational evidence supporting the activity of these peptides and gain new perspectives for peptide discovery.

**Electronic supplementary material:**

The online version of this article (doi:10.1186/s12859-016-1458-y) contains supplementary material, which is available to authorized users.

## Background

Recent advances in transcriptomics and proteomics have revealed a more complex transcriptome and proteome than formerly understood. In the past few years it has become increasingly clear that the short open reading frames (sORFs) embedded in intergenic regions, pseudogenes or non-coding RNAs (ncRNAs) can be directly translated into bioactive peptides [1]. These peptides may partake in an array of functions such as cellular signaling, as antibiotics, regulators of morphology, toxins/anti-toxins, chaperones, may stabilize protein complexes or serve as structural proteins [2–4]. However, genomic and functional annotation of novel sORFs in *Arabidopsis thaliana* is far from complete. To aid in annotation of peptide-encoding genes and deciphering their functions, dedicated resources to browse and access sORF-encoded peptides in *A. thaliana* would be very valuable. Several efforts have gone in this direction including Araport, a comprehensive information portal for plant biology research harbouring *A. thaliana*-related annotations and gene information [5], the Arabidopsis Unannotated Secreted Peptide Database, containing information on putative secreted peptides [6], and HanaDB-AT, providing transcriptome information in *A. thaliana* for annotated coding genes, ncRNA genes and sORFs [7, 8]. However, a comprehensive resource with all-inclusive information on peptides encoded by sORFs from *A. thaliana* is currently lacking. Therefore, we have developed a webserver named ARA-PEPs to provide the research community with up-to-date information on putative peptides in *A. thaliana*, collected from publicly available datasets and predicted based on novel expression data.

**Fig. 1:**
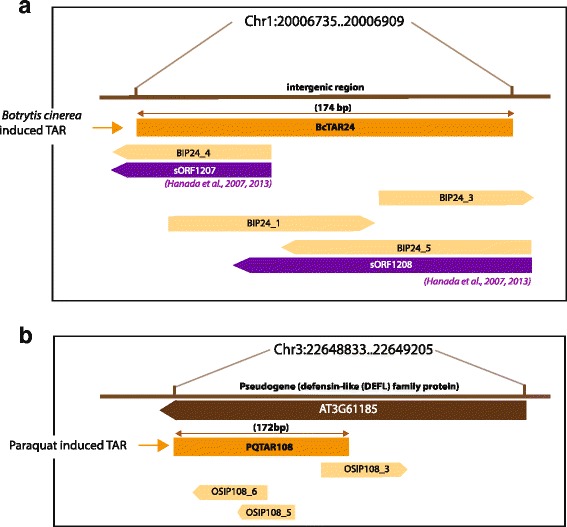
Overview of transcriptionally active regions (TARs) with reference to TAIR10 annotation that might encode stress-induced peptides (SIPs). SIPs are canonically translated (starting with AUG) sORFs of at least 10 amino acid (AA) length. A SIP can either be a *Botrytis cinerea* induced peptide (BIP) or an Oxidative stress induced peptide (OSIP) depending upon the stress applied in our study. **a** BcTAR24 harbors multiple sORFs in forward and reverse frames that might encode several BIPs. BIP24_4 has been annotated by Hanada et al., 2007, 2013 as sORF1207. Likewise, BIP24_5 has been annotated as sORF1208. **b** PQTAR108 lies within pseudogene AT3G61185 and harbors several sORFs in forward and reverse frames

Proper annotation of genes and other functionally relevant features is a major challenge in converting previously unknown genome sequences into resources that can be used by the research community. Recently, several tools such as CIPHER [9], GeneMarkS-T [10] and TransDecoder [11] from the Trinity package [12] have been developed to predict protein coding regions in RNA transcripts of eukaryotes. TransDecoder is a tool that identifies RNA transcripts generated by *de novo* transcriptome reconstruction methods such as Trinity or constructed based on RNA-seq alignments using TopHat-Cufflinks [13, 14] method. CIPHER uses a coding score metric to compute the coding potential of ORFs in sequences. GeneMarkS-T is used for ab initio gene finding and identification of translation initiation sites in eukaryotic genomes. These tools require a minimal ORF length to obtain a significant signal and are thus not very well suited for finding sORFs. In our study we have used an assortment of bioinformatics tools and in-house scripts to screen stress-induced peptides (SIPs) encoded by transcriptionally active regions (TARs) and to map these peptides to other publicly available peptide annotations. Homology to sequences in other plant genomes further supports the functionality of these peptides. The whole study aimed at enriching the existing pool of novel peptides encoded by sORFs in *A. thaliana*. The relationship between TARs, SIPs and other annotated sORFs listed in ARA-PEPs database is illustrated in Fig. 1a and b. The data is freely available online through the URL: http://www.biw.kuleuven.be/CSB/ARA-PEPs. The interface is easy to use and allows the user to query peptides by name, peptide sequence, Tiling array expression level and chromosomal regions including chromosome number and position, presence of certain features such as signal sequences, transmembrane (TM) domains and the rates of synonymous (*dS*) and nonsynonymous (*dN*) substitutions per site (*dN/dS*) ratio of the entire ARA-PEPs dataset as well as Tiling array- and RNA-seq-identified expression levels of the SIPs. Sequence searches are also possible through a Basic Local Alignment Search Tool (BLAST) [15] interface against all the sequences stored in the ARA-PEPs database while it is also possible to browse the peptides alongside genome annotations through the embedded JBrowse application [16]. By integrating other publicly available information together with in-house expression data on one centralized webserver, we aim to provide a user-friendly, simple yet, resourceful platform for accessing sORF-encoded plant peptides.

## Construction and content

### Data source

Data for Arabidopsis unannotated secreted database (SecretedPeptides) were obtained upon request [6] and only peptides with transcriptional evidence were retained. The dataset on sORFs was downloaded from the Gene Expression Omnibus (GEO) database [17]; accession: GSE34188) and from the supplementary information of the published report (Hanada et al., [7, 8]). Data on SIPs were obtained, as overviewed in Additional file 1: Figure S1, using analyses of *A. thaliana* leaves under both abiotic or biotic stress conditions. We earlier identified genes potentially encoding oxidative stress-induced peptides (OSIPs) in *A. thaliana* using a Tiling array approach on leaves treated with the herbicide Paraquat [18], and could retrieve these data from GEO database (accession: GSE49001). In the present study a similar Tiling array analysis was also performed on *A. thaliana* leaves after biotic stress caused by the fungal pathogen *Botrytis cinerea* (accession: GSE84002). *B. cinerea*-induced peptides (BIPs) of at least 10 amino acids were identified by six-frame translations of TARs. Additionally, in this study transcriptome analysis was performed on another set of *A. thaliana* leaves, after identical biotic and abiotic stress conditions, using a complementary RNA-seq approach (SRA accession : SRP080911). Both the Tiling array and RNA-seq data were subsequently analyzed with in-house scripts and the Tuxedo pipeline [14]. The OSIPs and BIPs are collectively called stress-induced peptides (SIPs).

### Tiling array analysis of biotic and abiotic stress data

Tiling array analysis, performed on mRNA extracted from Paraquat-treated *A. thaliana* leaves is described in De Coninck et al. [18]. Tiling array analysis on mRNA extracted from *A. thaliana* leaves collected 2 days post inoculation with the fungus *B. cinerea* was performed in a similar way (Additional file 2: Supplementary methods). The *B. cinerea* induced raw dataset have been deposited in GEO (accession: GSE84002).

### RNA-seq analysis of biotic and abiotic stress data

RNA-seq analysis was performed on mRNA extracted from *A. thaliana* leaves treated with Paraquat or *B. cinerea* (Additional file 2: Supplementary methods). A total of 334,624,105 reads were obtained from 48 samples which amounts to an average of 6971335.52 reads per sample (Additional file 3: Table S1). Raw sequencing reads have been deposited in SRA (study accession: SRP080911). Processed reads after quality control were mapped to *A. thaliana* genome. TopHat2 was used to align the reads against the TAIR10 reference genome using default parameters [13]. After running TopHat2, the resulting BAM files were provided to Cufflinks to generate a transcriptome assembly for each condition. These assemblies were then merged together using the Cuffmerge utility, which is included with the Cufflinks package [14]. This merged assembly provides a uniform basis for calculating gene and transcript expression in each condition. The reads and the merged assembly were fed to Cuffdiff, which calculated expression levels and tested the statistical significance of the observed changes. Transcript abundances are reported in FPKM (expected fragments per kilobase of transcript per million fragments sequenced). We used several plotting methods such as model fitting, assessment of FPKM distributions across samples etc. for quality-control or global analysis of the cufflinks data (Additional file 4: Figure S6). Finally the gene loci and isoforms identified using TopHat2 and Cufflinks was checked for overlap with the previously identified TARs from the Tiling array data using BEDTools utilities (Additional file 5: Figure S2; Additional file 6: Figure S7). CummeRbund was used to plot the results and visualize the expression data. For identification and calculation of expression levels of novel, unannotated, intergenic TARs we used Cufflinks-Cuffcompare-Cuffdiff methodology (Additional file 2: Supplementary methods; Additional file 5: Figure S2; Additional file 7: Figure S8).

### Conservation analysis of translated SIPs across multiple species

Using Tiling arrays, 195 TARs in the *B. cinerea* induced dataset and 176 TARs in the Paraquat induced dataset were identified. The overlap between the 2 datasets was merged and 290 unique TARs were re-labelled (Additional file 1: Figure S1). As the initial experiments were conducted using the TAIR7 genome assembly, the TARs were now mapped to the recent TAIR10 [19] genome assembly using Bowtie short read aligner [20]. The TARs identified by both Tiling arrays and RNA-seq analysis were pooled together for the conservation analysis. The nucleotide sequences were extracted from the TAIR10 genome using a custom R script and all 6-frame translations were carried out on the nucleotide sequences using Emboss Transeq (http://www.ebi.ac.uk/Tools/st/emboss_transeq/). Homologous sequences were screened using the similarity search algorithm tBLASTn [15] against 8 plant genomes. The genomes of *Oryza sativa japonica, Triticum aestivum, Zea mays, Hordeum vulgare, Solanum lycopersicum, Vitis vinifera, Brassica rapa* and *Arabidopsis lyrata* were downloaded from Ensembl through the FTP server (ftp://ftp.ensemblgenomes.org). The cutoff E-value was fixed at 0.001 and a 300 bp extra sequence was added on either side of the active region to not miss the chance of an in-frame start/stop codon (Additional file 8: Figure S4 a). In the first iteration of tBLASTn, the peptide sequences corresponding to homologous TARs were identified. Complete peptide sequences of at least 10 amino acids beginning with a canonical start codon and the sequence not truncated by a stop codon were extracted and used to run the second iteration of tBLASTn. Finally, homologous nucleotide and protein sequences were parsed out using the peptide positions from the tBLASTn output and taken for further analysis. Sequences overlapping with transposons and coding sequences were excluded.

### Scoring alignments of homologous SIPs

Homologous pairs were aligned using CLUSTALW2 [21]; all the pairwise alignments were scored using a BLOSUM80 scoring matrix and finally the mean of the pairwise alignments was computed. The mean alignment score was used as a measure to assess the quality of the alignments. We used the BLOSUM80 matrix instead of the default BLOSUM62 matrix in order to discriminate highly homologous sequences.

### Evaluating evolutionary pressure on the SIPs

In order to understand the dynamics of molecular sequence evolution, the rates of synonymous (*dS*) and nonsynonymous (*dN*) substitutions per site were estimated using PAML4.8 software [22]. The homologous peptides were identified (Additional file 8: Figure S4 b) and the nucleotide sequences of the homologs were extracted from the 8 plant genomes using functions from R seqinR package (http://seqinr.r-forge.r-project.org/). The sequences were aligned using CLUSTALW2 using a default substitution matrix (BLOSUM62). The Gap Opening Penalty was set to default = 10 and the Gap Extension Penalty was set to 1. Using PAL2NAL.v14 [23] codon alignments were obtained after removal of gaps and in frame stop codons in the alignment files. The codeml program from PAML was used to calculate the synonymous and nonsynonymous substitution rates. The detailed parameters for estimating the *dN/dS* score using codeml is explained in Additional file 2: Supplementary methods.

### Mapping TARs to other public datasets

The TARs were mapped to recent annotations in TAIR10. We also checked if the TARs overlap with any annotated ncRNAs such as in PLncDB. PLncDB currently deposits lncRNA information of several *A. thaliana* ncRNAs identified using Reproducibility based Tiling array Analysis Strategy (RepTAS), Tiling arrays, RNA-seq and EST analysis [24]. The gff3 files were downloaded from TAIR10 and PLncDB, the nucleotide sequences were parsed and overlaps between the SIPs and the annotated datasets were determined using intersectBed from the BEDTools utilities [25].

### Functional analyses of the ARA-PEPs database

In order to assign functional roles to the peptides, data was collected across several resources. The sORFs/peptides predicted by other research groups were collected and pooled together (Hanada et al., [7, 8]; Lease and Walker, [6]). In the first step of analysis, the set of Cysteine-rich peptides (CRPs) and Defensin-like (DEFL) genes were downloaded [26–29] and the Pfam database was downloaded from (http://xfam.org) [30]. hmmsearch from HMMER-3 [31] package was used to iteratively search the putative peptides against these databases by means of a generous E-value of 10. The hits were piled and the search was iterated again using the new hit set and the E-value set to 0.01. We used Pfam2GO annotations for the domain matches (http://www.geneontology.org/external2go/pfam2go). This is an automated mapping of GO terms to Pfam domains. Pfam2GO annotations are not available for all the domains. Standalone version of SignalP [32] was used to predict cleavage site of signal sequences, TMHMM [33] was used to predict TM regions. We used the ScanProsite tool (http://prosite.expasy.org/scanprosite/) for detecting PROSITE signature matches [34]. PROSITE database consists of a large collection of biologically meaningful signatures that are described as patterns (regular expressions), used for short motif detection, or generalized profiles (weight matrices) for sensitive detection of larger domains. Additionally, the ELM database which is a repository of eukaryotic linear motifs (http://elm.eu.org/) was used to detect functional sites in the putative peptides [35]. As the motifs are very short we correlated the findings with sequence conservations.

### Finding potential peptide clusters in the ARA-PEPs database

The Markov Cluster Algorithm (mcl) was used to group all the peptide sequences in ARA-PEPs into putative clusters [36]. All the peptide sequences collected across different sources were pooled together and the similarity between all pairs of sequences were determined by “all-by-all” using BLASTP 2.2.28+ algorithm at E-value 0.001. We used the standard set of values viz, 1.4, 2 and 3 for the (−I) option which regulates cluster granularity (Additional file 9: Figure S5; Additional file 2: Supplementary methods).

### Database design and implementation as a webserver

Resulting tables from the data processing pipeline were loaded into a MySQL database and were normalized to remove data redundancy (Additional file 5: Figure S2). The webserver was built in a Linux, Apache2, PHP5 and MySQL5 environment. The ARA-PEPs front-end layer uses HTML5, Bootstrap CSS library (http://getbootstrap.com/), JavaScript and jQuery (https://jquery.com/). ARA-PEPs hosts JBrowse-1.12-0 which is a dynamic and fast genome browser that integrates gene structure and genomic attributes, expression data, etc. We have also embedded other JavaScript visualization tools such as pVIz.js, a protein feature viewer for visualization of TM domains [37] and JavaScript Sequence Alignment Viewer (JSAV) [38] for visualization of alignments from our bioinformatics analysis. Interactive charts from http://www.highcharts.com/ have been integrated to visualize the data. These tools are compatible with any browser without the need of any external plugins. The web application has been successfully tested on Chrome 49, Firefox 41.0.2, Opera 28, Safari 9.1, Internet Explorer 11.

## Utility and discussion

We have generated ARA-PEPs, a repository of peptides encoded by sORFs of *A. thaliana* with transcript evidence.

### Data content

**Fig. 2:**
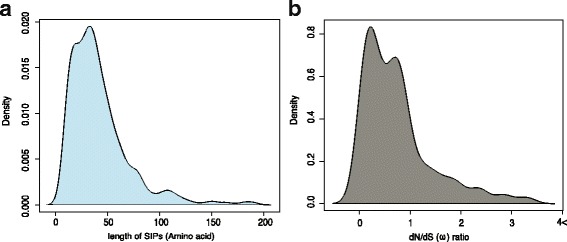
**a** Distribution of length of SIPs. Median length = 35 AA. **b** Distribution of estimated selective pressure of conserved SIPs

**Fig. 3:**
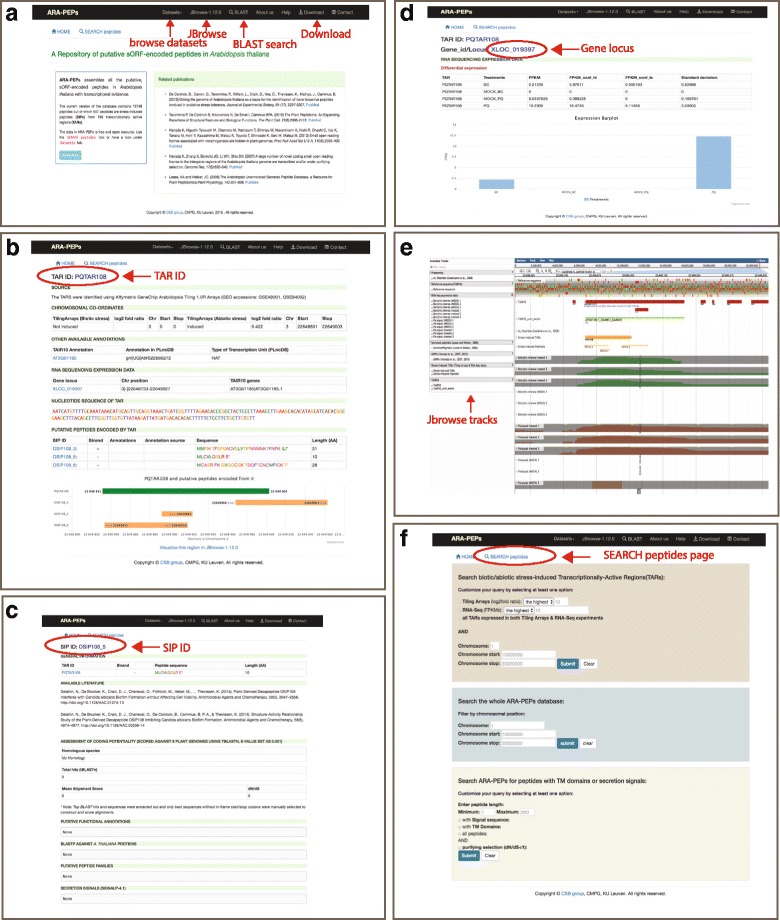
Overview of the ARA-PEPs website. **a** home page. **b** Screenshot of a PQTAR108 induced by abiotic stress (oxidative stress by the herbicide Paraquat). **c** Screenshot of detailed information of peptide OSIP108_5 (or OSIP108) induced by abiotic stress (oxidative stress by the herbicide Paraquat). **d** Page showing differential expression of locus XLOC_019397 corresponding to PQTAR108. **e** Screenshot of JBrowse-1.12.0 embedded in the ARA-PEPs webserver showing PQTAR108, OSIP108_3, OSIP108_5 and OSIP108_6 alongside other available annotations and expression levels from RNA-seq data. **f** the “SEARCH peptides” page where users can customize their query

ARA-PEPs currently contains 5240 ‘SecretedPeptides’, 7901 ‘sORFs’ and 607 ‘SIPs’ of which 341 are novel. No peptides were commonly identified in all three datasets, and only a small number of peptides were predicted in at least two out of three datasets, showing that the approaches are largely complementary (Additional file 10: Figure S3). Functional analysis was carried out using an assortment of bioinformatics tools and the results provide information that these peptides might have specific features, functional roles or may be grouped together as specific peptide families (Additional file 5: Figure S2). The novel SIPs dataset has been generated based on both Tiling array and RNA-seq analysis as detailed in Additional file 1: Figure S1 leading to 607 putative SIPs encoded by 189 TARs of which 100 are novel. From TARs identified by Tiling arrays, 91 out of 144 were also induced in RNA-seq dataset thus confirming that these regions are indeed transcribed (Additional file 6: Figure S7). We identified 45 new intergenic TARs from the RNA-seq data (Additional file 7: Figure S8). The median length of the SIPs is 35 amino acids (Fig. 2a). Sequence analysis identified 279 peptides with sequence similarity with other plant species and 159 peptides with *dN/dS* < 1 (Fig. 2b, Additional file 1: Figure S1, Additional file 8: Figure S4). Mapping TARs to different annotations in PLncDB revealed that some peptides lie within annotated ncRNAs (Fig. 3b). Clustering of peptides across the three datasets in ARA-PEPs based on sequence similarity resulted in several large clusters (>50 peptides), suggesting the existence of some peptide groups with common molecular functionalities (Additional file 9: Figure S5). We also found 5430 peptides with secretion signals and 2510 peptides with TM domains across the three datasets (Additional file 5: Figure S2).

### Search and download features

ARA-PEPs is a comprehensive webserver for searching, browsing, visualizing and downloading sORF-encoded peptides. In the ARA-PEPs webserver, the ‘SEARCH peptides’ tool enables queries on several criteria, including presence of signal sequences, TM domains and *dN/dS* ratio of the entire ARA-PEPs dataset, chromosome number and position as well as Tiling array- and RNA-seq-identified expression levels of the SIPs (Fig. 3f). It is also possible to browse just through the list of SIPs, sORFs (Hanada et al., [7, 8]) or SecretedPeptides (Lease and Walker, [6]) by clicking on the ‘Datasets’ tab on the toolbar and selecting any of the above the peptide datasets (Fig. 3a). Clicking on a TAR (TAR ID) from the ‘SIPs’ dataset for example PQTAR108 will open up a page with detailed information about the TAR such as data source, chromosomal co-ordinates, nucleotide sequence of the TAR, other available annotations in TAIR10 and PLncDB, RNA-seq expression data etc. (Fig. 3b). From the TAR page the user can browse the RNA-seq expression levels of the TAR by clicking on the gene locus (XLOC_019397) (Fig. 3d). Clicking on any putative peptide (SIP ID) encoded by the TAR for example OSIP108_5 on the TAR page will yield information about the peptide such as any available literature, coding potentiality, functional annotations, putative peptide families, secretion signals, TM domains if any (Fig. 3c). We have provided a bulk download functionality to download all the peptide sequences in FASTA format or the genomic positions in BED format in the ARA-PEPs database (Fig. 3a). It is also possible to export all tabular data displayed in ARA-PEPs database as a csv file.

### Integrated tools for visualization

ARA-PEPs offers an interface to do a BLAST [15] search against the whole ARA-PEPs database using any user-uploaded sequence. The BLAST interface is accessible from the toolbar on the home page (Fig. 3a). ARA-PEPs also uses JBrowse-1.12-0 [16] to visualize genomic locations of peptides collected across different sources along the TAIR10 annotation (Fig. 3a and e). Below the reference sequence axis, the browser presents a stack of ‘tracks’ representing the TAIR 10 gene models. We have currently embedded several tracks, including the stress-induced TARs, SIPs, peptides determined by mass spectrometry [39], sORFs [7, 8], SecretedPeptides [6], TAIR10 unconfirmed exons and BAM data from the in-house RNA-seq analysis. The user can view these tracks via the hierarchical track selector on the left hand panel of the browser. JBrowse-1.12.0 also offers bulk download of interval-specific track data in common file formats (FASTA, GFF3, BED). JavaScript visualization tools such as pVIz.js, and JavaScript Sequence Alignment Viewer (JSAV) have been integrated to provide visualization of TM domains and raw alignments from our bioinformatics analysis respectively.

## Conclusions

We have constructed a comprehensive repository for browsing and visualizing sORF-encoded peptides in *A. thaliana* by combining publicly available and newly generated Tiling arrays and RNA-seq data in response to biotic/abiotic stress. ARA-PEPs is by far the largest repository of putative sORF-encoded putative peptide database with transcript evidence. The database provides external links to other databases thus facilitating the user to view other existing details about these peptide sequences. Integration of JBrowse and BLAST search will help biologists to comparatively access all available annotations pertaining to the sequences. The entire set of analyzed peptides in the current study can be downloaded from the download page of the webserver. Moreover, in future we intend to add more data from transcriptome analysis. In conclusion, the repository we generated here provides the basis of future studies on bioactive plant peptides, as part of the booming research on the plant peptidome, thereby aided by the accessibility and user-friendliness of the corresponding webserver.

## Availability and requirements

ARA-PEPs is available from http://www.biw.kuleuven.be/CSB/ARA-PEPs. The web server was built in a Linux, Apache2, PHP5 and MySQL5 environment. The ARA-PEPs front-end layer uses HTML5, Bootstrap CSS library (http://getbootstrap.com/), JavaScript and jQuery (https://jquery.com/). These tools are compatible with any browser without the need of any external plugins. The web application has been successfully tested on Chrome 49, Firefox 41.0.2, Opera 28, Safari 9.1, Internet Explorer 11. Copyright 2016 KU Leuven Computational Systems Biology, distributed under MIT license.

## Additional files

Additional file 1: Figure S1. Workflow for screening of stress-induced peptides (SIPs) in *A. thaliana*. The number of peptides passing through each step is listed. (PDF 891 kb)
Additional file 2: Supplementary methods. (DOC 59 kb)
Additional file 3: Table S1. Yield statistics table showing the number of bases, number of reads and read length after read pre-processing. (XLS 28 kb)
Additional file 4: Figure S6.
**a**, shows the distributions of log_10_ FPKM scores across samples. **b**, Plot showing counts vs dispersion. This plot estimates overdispersion for the 4 samples. **c**, Squared coefficient of variation (CV^2^) of genes as a function of expression level (log_10_ FPKM) for the different samples. **d**, Squared coefficient of variation (CV^2^) of isoforms as a function of expression level (log_10_ FPKM) for the different samples. The plots show the degree of variability of genes and isoforms for the different samples. Squared coefficient of variation is a normalized measure of cross-replicate variability that can be useful for evaluating the quality of the RNA-seq data. (PDF 2082 kb)
Additional file 5: Figure S2. Pipeline showing an overview of the different tools/methods used for data integration in ARA-PEPs. (PDF 852 kb)
Additional file 6: Figure S7. Heatmap displaying 96 loci (corresponding to 91 TARs) that are assembled as transcripts by Cufflinks and differentially expressed between biological replicates of untreated samples (MOCK_PQ and MOCK_BC) and treatment groups (PQ and BC). (PDF 923 kb)
Additional file 7: Figure S8. Heatmap displaying 45 new intergenic TARs from the RNA-seq data, that are differentially expressed between untreated samples (MOCK_PQ and MOCK_BC) and treatment groups (PQ and BC). (PDF 817 kb)
Additional file 8: Figure S4.
**a**, Scatterplot showing dependency of log_10_ (query coverage) on the log_10_ (Bit score) of all the tBLASTn hits. The heatmap shows the low density hits in orange and the high density hits in purple. The hits with high coverage and high Bit Score were taken for further analysis. **b**, Scatterplot showing dependency of log_10_ (query coverage) on the log_10_ (Bit score) of all the tBLASTn hits with Bit score > 100 and grouped by genomes. These hits were taken for estimation of *dN/dS* ratio. (PDF 5709 kb)
Additional file 9: Figure S5. Scatterplot showing the frequency of peptide clusters against cluster sizes obtained after using different mcl Inflation thresholds (1.4,2,3) on a logarithmic scale. (PDF 899 kb)
Additional file 10: Figure S3. Overlap between the 3 datasets in the ARA-PEPs database. (PDF 883 kb)

## Abbreviations

BIPs: *Botrytis cinerea*-induced peptides
BLAST: Basic local alignment search tool
CRPs: Cysteine-rich peptides
DEFL: Defensin-like genes
*dN/dS*: The ratio of nonsynonymous and synonymous substitutions per site
ncRNAs: non-coding RNAs
OSIPs: Oxidative stress-induced peptides
SIPs: Stress-induced peptides
sORFs: small open reading frames
TARs: Transcriptionally active regions
