# Supplementary figures and images for: ARA-PEPs: a repository of putative sORF-encoded peptides in Arabidopsis thaliana
Source: BMC Bioinformatics. 2017 Jan 17;18:37. doi: 10.1186/s12859-016-1458-y (PMC5240266; doi:10.1186/s12859-016-1458-y)

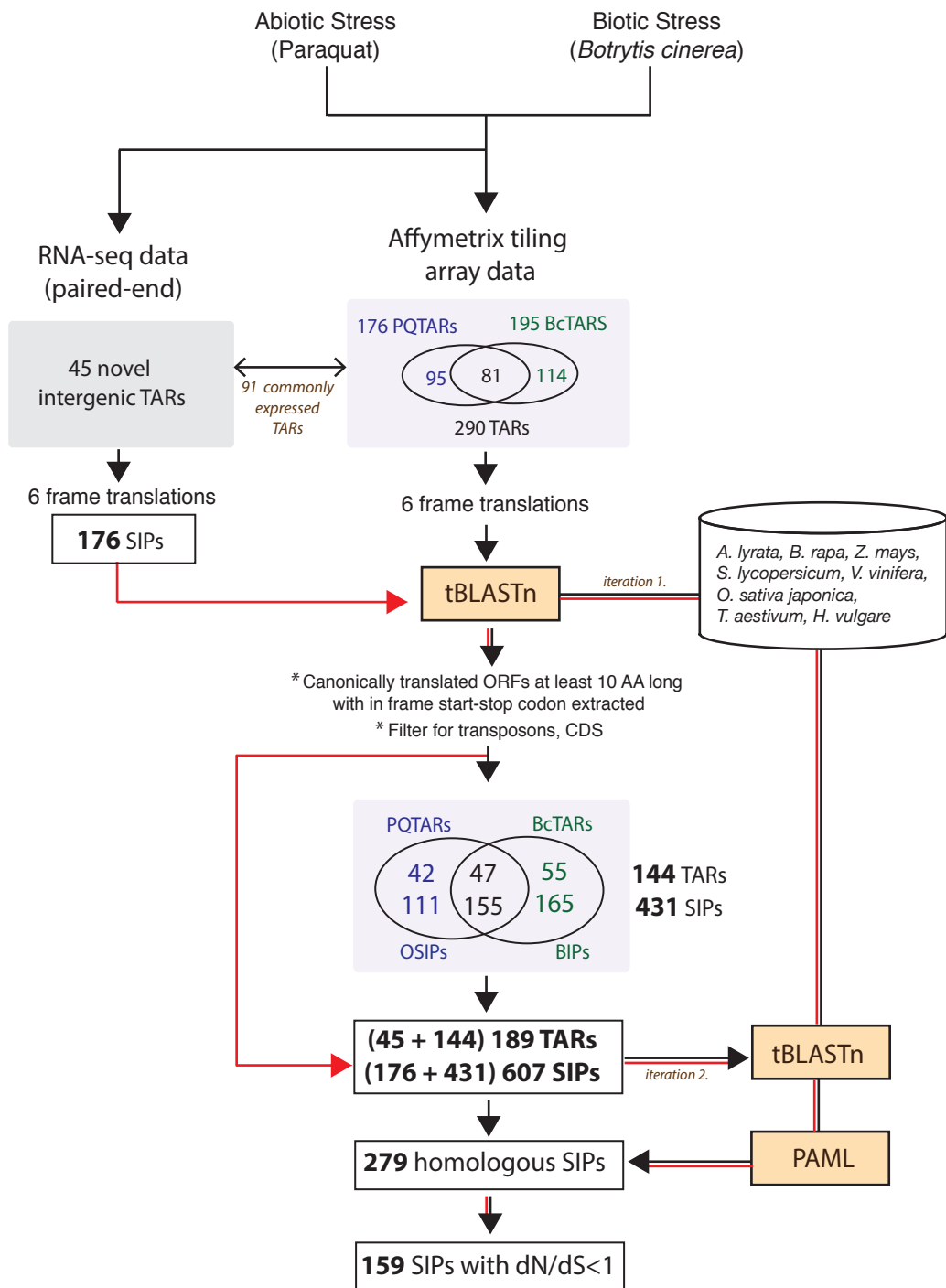

Supplement: Additional file 1: Figure S1. — Workflow for screening of stress-induced peptides (SIPs) in A. thaliana. The number of peptides passing through each step is listed. (PDF 891 kb) [file 12859_2016_1458_MOESM1_ESM.pdf]

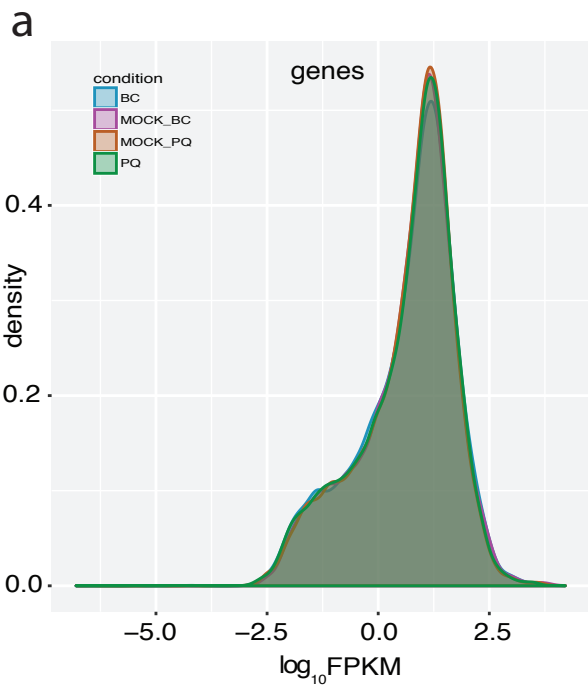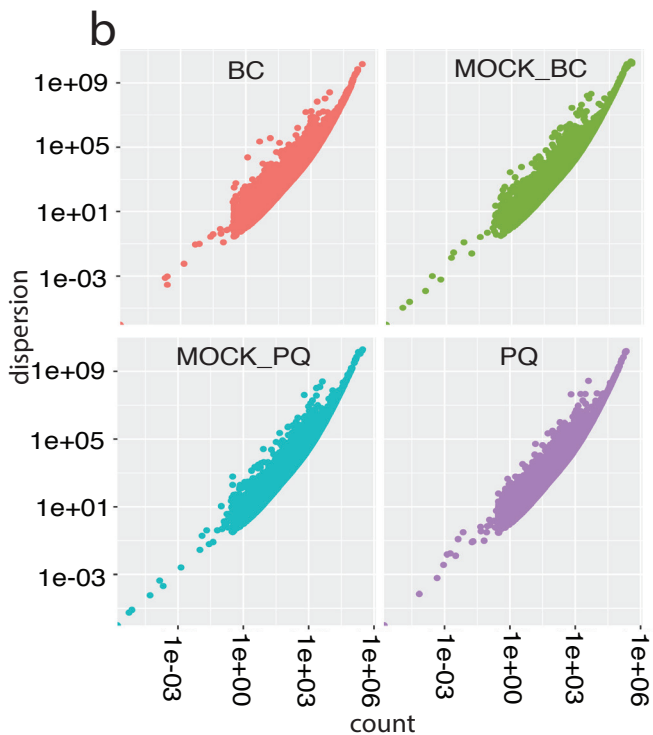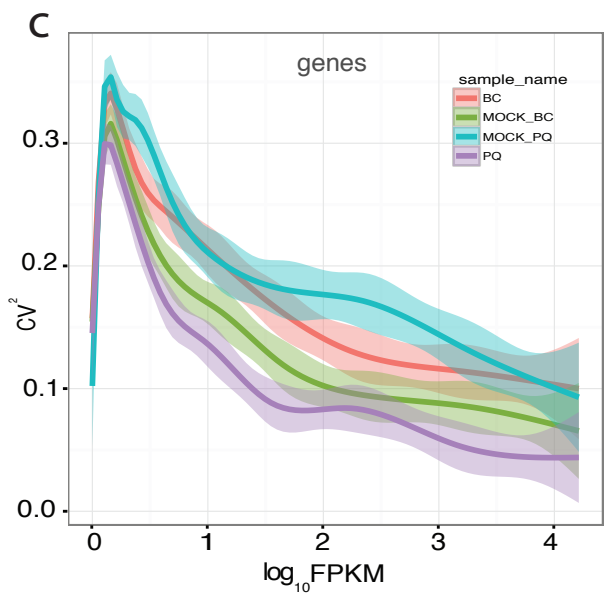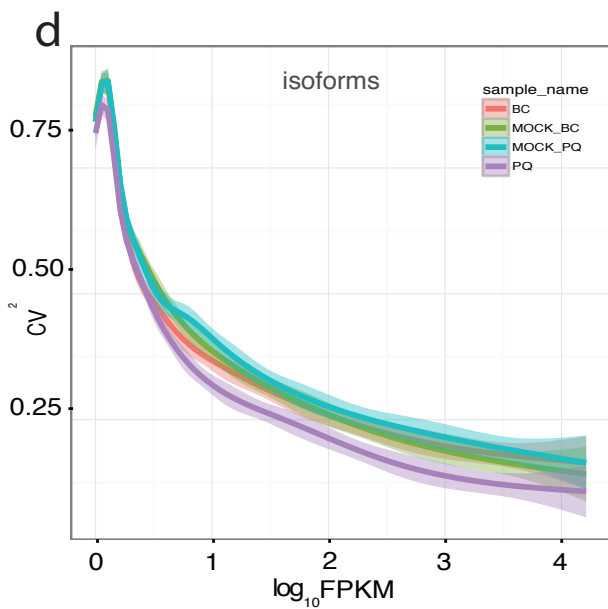

Supplement: Additional file 4: Figure S6. — a, shows the distributions of log10 FPKM scores across samples. b, Plot showing counts vs dispersion. This plot estimates overdispersion for the 4 samples. c, Squared coefficient of variation (CV2) of genes as a function of expression level (log10 FPKM) for the different samples. d, Squared coefficient of variation (CV2) of isoforms as a function of expression level (log10 FPKM) for the different samples. The plots show the degree of variability of genes and isoforms for the different samples. Squared coefficient of variation is a normalized measure of cross-replicate variability that can be useful for evaluating the quality of the RNA-seq data. (PDF 2082 kb) [file 12859_2016_1458_MOESM4_ESM.pdf]

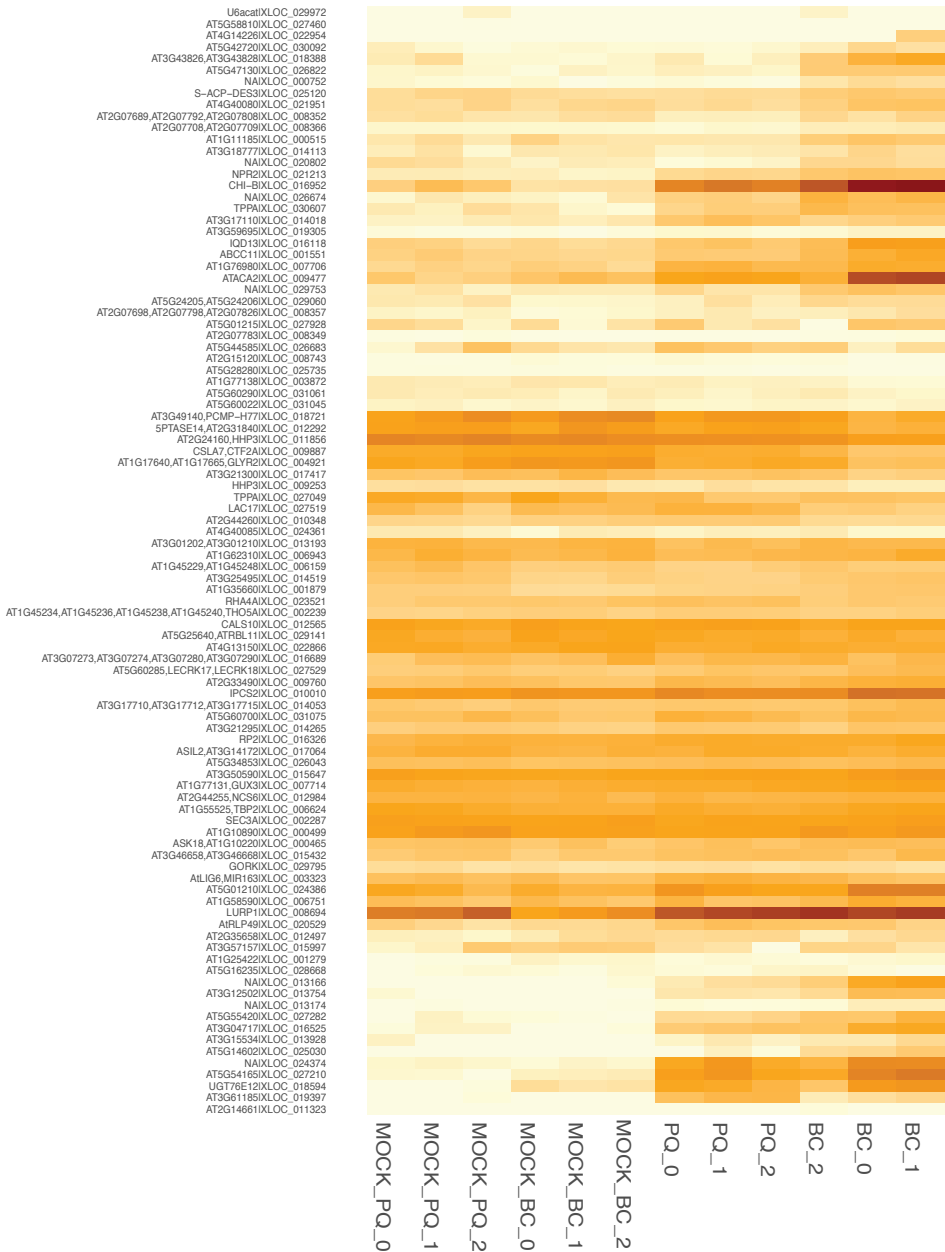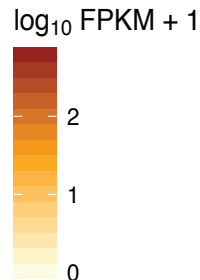

Supplement: Additional file 6: Figure S7. — Heatmap displaying 96 loci (corresponding to 91 TARs) that are assembled as transcripts by Cufflinks and differentially expressed between biological replicates of untreated samples (MOCK_PQ and MOCK_BC) and treatment groups (PQ and BC). (PDF 923 kb) [file 12859_2016_1458_MOESM6_ESM.pdf]

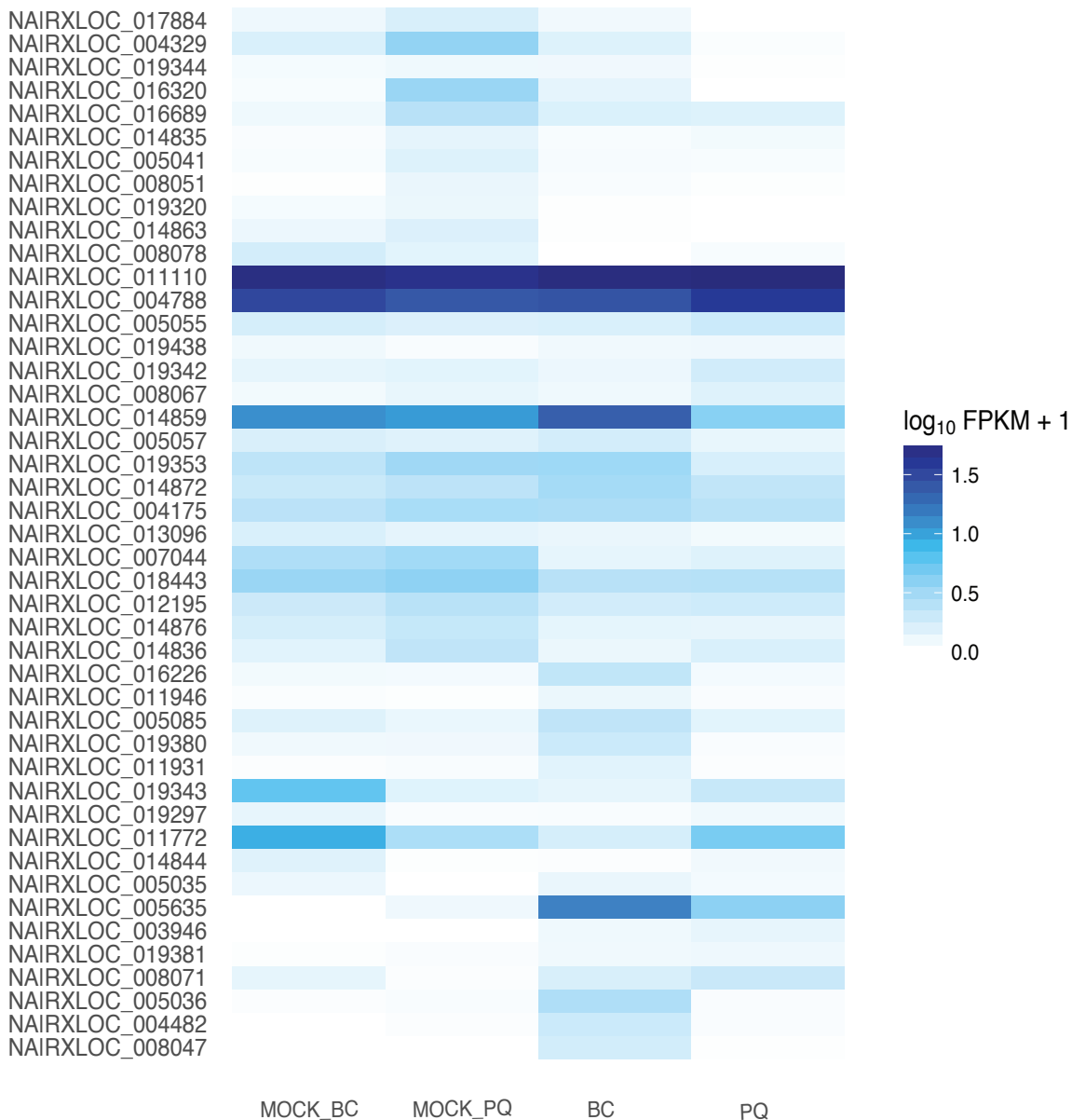

Supplement: Additional file 7: Figure S8. — Heatmap displaying 45 new intergenic TARs from the RNA-seq data, that are differentially expressed between untreated samples (MOCK_PQ and MOCK_BC) and treatment groups (PQ and BC). (PDF 817 kb) [file 12859_2016_1458_MOESM7_ESM.pdf]

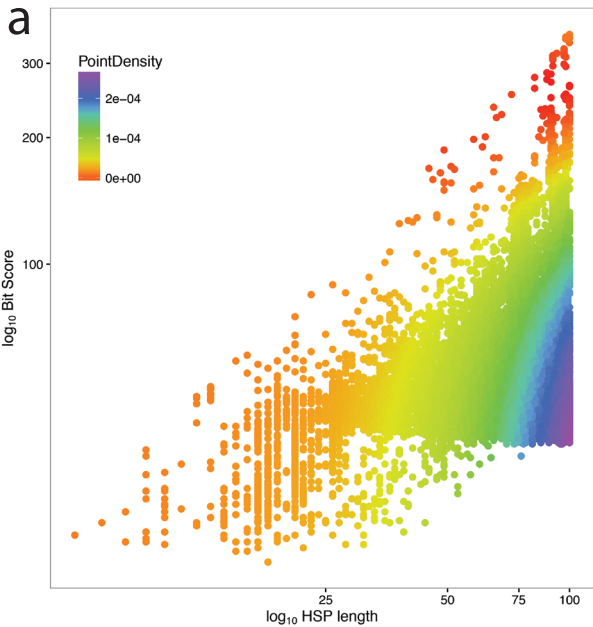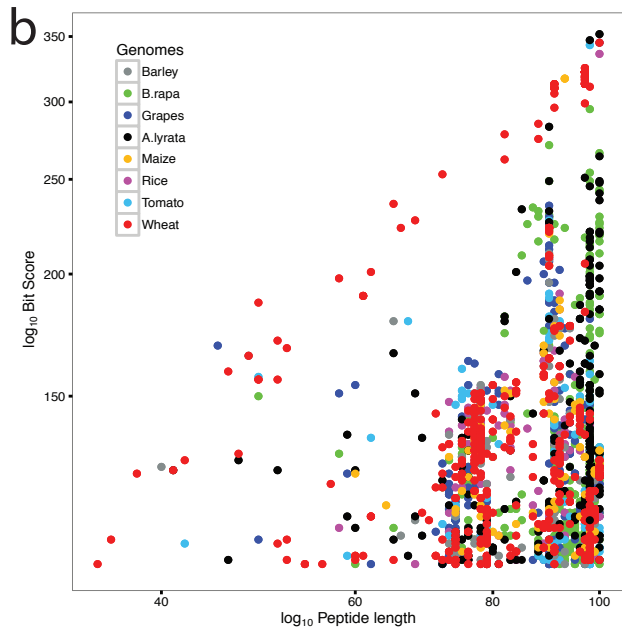

Supplement: Additional file 8: Figure S4. — a, Scatterplot showing dependency of log10 (query coverage) on the log10 (Bit score) of all the tBLASTn hits. The heatmap shows the low density hits in orange and the high density hits in purple. The hits with high coverage and high Bit Score were taken for further analysis. b, Scatterplot showing dependency of log10 (query coverage) on the log10 (Bit score) of all the tBLASTn hits with Bit score > 100 and grouped by genomes. These hits were taken for estimation of dN/dS ratio. (PDF 5709 kb) [file 12859_2016_1458_MOESM8_ESM.pdf]

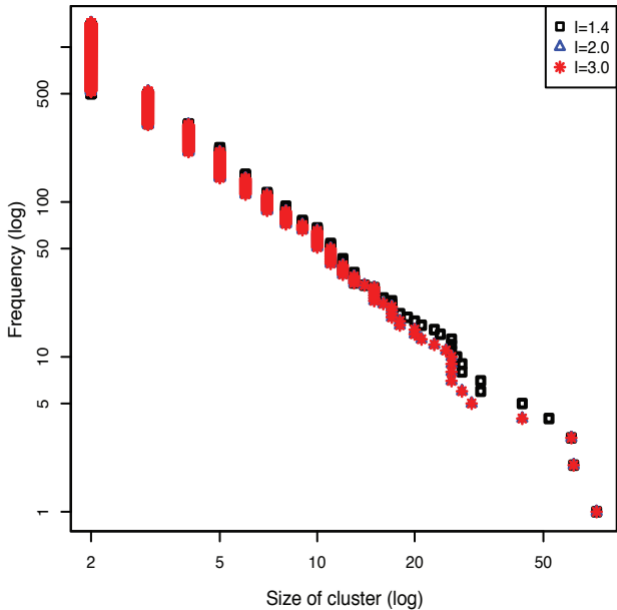

Supplement: Additional file 9: Figure S5. — Scatterplot showing the frequency of peptide clusters against cluster sizes obtained after using different mcl Inflation thresholds (1.4,2,3) on a logarithmic scale. (PDF 899 kb) [file 12859_2016_1458_MOESM9_ESM.pdf]

SecretedPeptides  
(Lease & Walker, 2006)

sORFs  
(Hanada et al., 2007, 2013)

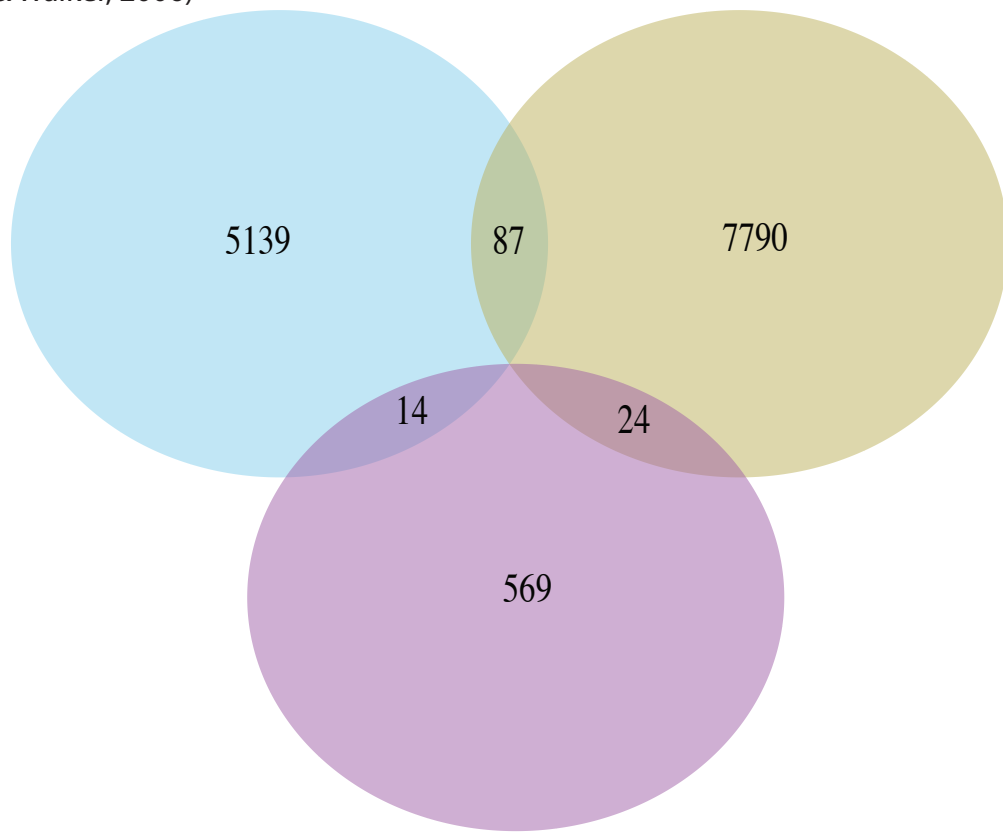

SIPs  
(De Coninck et al., 2013; current manuscript)

Supplement: Additional file 10: Figure S3. — Overlap between the 3 datasets in the ARA-PEPs database. (PDF 883 kb) [file 12859_2016_1458_MOESM10_ESM.pdf]
