# Supplementary material for: ARA-PEPs: a repository of putative sORF-encoded peptides in Arabidopsis thaliana
Source: BMC Bioinformatics. 2017 Jan 17;18:37. doi: 10.1186/s12859-016-1458-y (PMC5240266; doi:10.1186/s12859-016-1458-y)
Supplement: Additional file 2: — Supplementary methods. (DOC 59 kb) [file 12859_2016_1458_MOESM2_ESM.doc]

**Supplementary methods:**

**Plant material, treatments and identification of probes for tiling array analysis of biotic stress data**

In order to identify novel *Botrytis cinerea*-induced peptides (BIPs) in *Arabidopsis thaliana* a tiling array analysis (GeneChip® Arabidopsis Tiling 1.0R Arrays) was performed on mRNA extracted from *A. thaliana* leaves collected 2 days post inoculation with the fungus *B. cinerea* (BC). The experimental setup comprised of 2 replicates for *Botrytis cinerea* treated *A. thaliana* leaves and 2 replicates for control (water) treated *A. thaliana* leaves resulting in a total of 4 samples. Background correction, log2 transformation and interslide normalization were performed with the Affymetrix Tiling Analysis Software - Version 1.1, Build 2. Since the tiling arrays were based on the *A. thaliana* genome sequence of the 2007’s version of the TAIR database, the corresponding TAIR7 genome annotation was used for the analysis of the tiling array data.

The initial step of the Tiling Array experiment was the identification of probes that are part of transcriptional active regions (TARs), based on the probe intensities. In the first step, in order to identify transcriptionally active probes, the average log2 intensities of biological replicates were calculated and a threshold value of 8.8 was chosen to distinguish the probe intensities of TARs (ON probes) from the background signal (OFF probes). This threshold was determined by analyzing intensities of annotated genes that were known to be transcriptionally active. Secondly, groups of 4-13 successive transcriptionally active probes were combined into short transcriptionally active regions (TARs). Selection of smaller regions beyond the threshold of 4-13 successive probes would lead to a high number of false positives and towards the identification of very small genes that would be difficult to amplify at a later stage. We found that all active regions of 14-15 successive probes resulted in TARs that were previously annotated (TAIR7), supporting our choice of size limits. In a third step, TARs were withheld if the number of transcriptionally active probes was smaller in the control than in the BC treatment and if the average log2 intensity of all probes in the smallest of the two regions was at least 1.4 higher in the BC treatment than in the control. Both rules were based on the tiling array results for genomic regions that were known to be induced upon BC treatment. The former rule was added because of the frequent occurrence of probes having zero intensity in one of the four hybridizations. In small regions these zero values can have substantial influence on the average log2 intensity leading to false positive calls. The threshold for upregulation by BC was refined by comparing qRT-PCR and tiling array results for 30 selected TARs. All upregulations of 1.4 or more (corresponding to 2.6 fold or more) in the tiling arrays could be confirmed by qRT-PCR supporting our choice of threshold. Finally, each selected region was compared to the *A. thaliana* TAIR7 genome annotation and only regions that were located in intergenic regions, introns or pseudogenes were withheld [1].

**Plant material, treatments and analysis of biotic and abiotic stress RNA-seq data**

RNA-seq analysis was performed on mRNA extracted from *A. thaliana* leaves treated with Paraquat or *B. cinerea*. To this end, plants were sprayed evenly until run off with Paraquat (25 μM; Sigma) or mock treated with sterile water. For the inoculation with *B. cinerea,* plants were sprayed evenly until run off with a spore suspension of 5 x 10^4^ *B. cinerea* B05.10 spores/ml in 1/2 PDB (12 g/L Potato Dextrose Broth) or mock treated with ½ PDB. Plants were kept at 100% relative humidity. Two days post treatment, a pool of six randomly selected plants was collected and all aboveground plant material was harvested in liquid nitrogen and stored at -80°C prior to further analysis. Three biological replicates were used for both the control plants and treated plants. Total RNA was isolated using the Plant RNA Qiagen RNeasy kit according to the manufacturer’s instruction (Qiagen,Valencia, CA, USA). RNA quantity and quality control was performed using a NanoDrop ND-1000 spectrophotometer (NanoDrop Technologies, DE, USA) and the Agilent 2100 Bioanalyzer (Agilent Technologies, CA, USA). Library preparation was performed using the Illumina (USA) TruSeq kit and 150 bp paired-end sequencing was performed in one lane of an Illumina NextSeq 500 sequencer using the NextSeq High Output kit (300 cycles, Illumina, USA) according to the manufacturer’s instructions. Each biological replicate had further 4 technical replicates in different lanes, resulting in a total of 48 samples. A total of 334,624,105 reads were obtained from 48 samples which amounts to an average of 6971335.52 reads per sample (Additional file 10: Table S1). The samples were named based on their replicate number and their experimental condition. In the manuscript, the untreated samples are referred to as MOCK_PQ and MOCK_BC and the treated samples as PQ and BC respectively.

Low quality ends (< Q20) and adapters were trimmed and reads shorter than 35 bp after trimming were removed. Poly-A reads (more than 90% of the bases equal A), ambiguous reads (containing N), low quality reads (more than 50% of the bases < Q25) and artefact reads (all but 3 bases in the read equal one base type) were removed. We carried out FastQC analysis to check the quality of the reads after the read pre-processing step (<http://www.bioinformatics.babraham.ac.uk/projects/fastqc/>).

TopHat2[2] was used to align the reads against the TAIR10 reference genome using default parameters and downstream analysis of the RNA-seq data was carried out using the Tuxedo protocol [3]. In order to detect novel, unannotated TARs we used the utility program called 'Cuffcompare' from the Cufflinks package that can compare Cufflinks assemblies to reference annotation files and help sort out novel genes from known ones. Percentage of loci classified in each Cufflinks class code after running cuffcompare on the RNA-seq dataset was examined. A transcript is deemed novel if it does not overlap any known transcript in TAIR10 and has a class code “u”, “x”, “I”, “j” in the output of Cuffcompare (i.e., unknown intergenic transcript, cis-antisense, intronic, alternatively spliced novel isoform respectively). We extracted out transcripts with class code “u” and  less than 1000 bp in length.

Additionally, we also converted the mapped BAM results to gene level counts for each sample and finally merged all resulting count tables into a global table. We used the HTSeq-count [4] utility to compute gene counts. HTseq-count *-m* option implies the mode to handle reads overlapping more than one feature and it was set to intersection-strict, *-s* implies whether the data is from a strand-specific assay and it was set as no, *-i* option or gtf/gff attribute to be used as feature id was set as gene_id and *-a* option was set to 10 directing htseq-count to skip reads with alignment score less than 10 and the option *-t* or feature type to be used was set as exon, meaning that all features of other type are ignored. All other reads mapped to intergenic and/or intronic regions will contribute to “no_feature”, “ambiguous” in the output file.

**Detailed parameters for estimating *dN/dS* ratio of SIPs using PAML4.8**

The codeml program from PAML[5] was used to calculate the synonymous and nonsynonymous substitution rates using the one ratio model M0. The CodonFreq option uses nucleotide frequencies at the different codon positions to estimate overall codon frequencies. It was set at 2:F3X4. The *NSsites* option implies whether ω should be allowed to vary among sites. The *model* option implies whether ω should be allowed to vary among branches in the tree. We run the analysis with model M0 (both the *NSsites* and *model* option set to 0) for all branches of the tree meaning that a single ω was allowed for all the codon sites and all its branches. Apart from M0, we also tested the other models M1 (nearly neutral) and M2 (positive selection) on the same data. The runmode was set to “0” (the user-defined input tree) for sites and lineage variation analyses. For pairwise estimates the runmode was set to “-2” and no treefile was required.

**Parameters for finding potential peptide clusters using mcl algorithm in the entire ARA-PEPs database**

The Markov Cluster Algorithm (mcl) was used to group all the peptide sequences in ARA-PEPs into putative clusters [6]. All the peptide sequences collected across different sources were pooled together and the similarity between all pairs of sequences were determined by “all-by-all” using BLASTP 2.2.28+ algorithm at E-value 0.001. The external columnar BLAST output was transformed to an ABC-formatted file called “seq.abc”. Using *mcxload*, the abc formatted file was loaded, a network file “seq.mci” and a dictionary file “seq.tab” were generated as outputs. The --stream-mirror option sets the resulting network as undirected. The next option, --abc-neg-log10 transforms the input BLAST E-values by taking the logarithm in base 10 and subsequently negating the sign. First we generated an abstract clustering representation using *mcl* from which the labeled output was obtained using *mcxdump*. The main adjustment for changing inflation is the -I option and it also regulates cluster granularity. More specifically increasing the value of -I will increase cluster granularity. We used the standard set of values *viz*, 1.4, 2 and 3.

**References**

1. De Coninck B, Carron D, Tavormina P, Willem L, Craik DJ, Vos C, et al. Mining the genome of Arabidopsis thaliana as a basis for the identification of novel bioactive peptides involved in oxidative stress tolerance. J. Exp. Bot. [Internet]. 2013 [cited 2015 Jan 29];64:5297–307. Available from: http://jxb.oxfordjournals.org/content/64/17/5297.long

2. Trapnell C, Pachter L, Salzberg SL. TopHat : discovering splice junctions with RNA-Seq. 2009;25:1105–11.

3. Trapnell C, Roberts A, Goff L, Pertea G, Kim D, Kelley DR, et al. Differential gene and transcript expression analysis of RNA-seq experiments with TopHat and Cufflinks. Nat. Protoc. [Internet]. 2012;7:562–78. Available from: http://www.pubmedcentral.nih.gov/articlerender.fcgi?artid=3334321&tool=pmcentrez&rendertype=abstract

4. Anders S, Pyl PT, Huber W. HTSeq – A Python framework to work with high-throughput sequencing data HTSeq – A Python framework to work with high-throughput sequencing data. Bioinformatics. 2014;31:0–5.

5. Yang Z. PAML 4: Phylogenetic analysis by maximum likelihood. Mol. Biol. Evol. 2007;24:1586–91.

6. van Dongen S. Graph clustering. Graph Stimul. by flow Clust. 2000;PhD thesis:University of Utrecht.
