# Supplementary material for: ARA-PEPs: a repository of putative sORF-encoded peptides in Arabidopsis thaliana
Source: BMC Bioinformatics. 2017 Jan 17;18:37. doi: 10.1186/s12859-016-1458-y (PMC5240266; doi:10.1186/s12859-016-1458-y)
Supplement: Additional file 5: Figure S2. — Pipeline showing an overview of the different tools/methods used for data integration in ARA-PEPs. (PDF 852 kb) [file 12859_2016_1458_MOESM5_ESM.pdf]

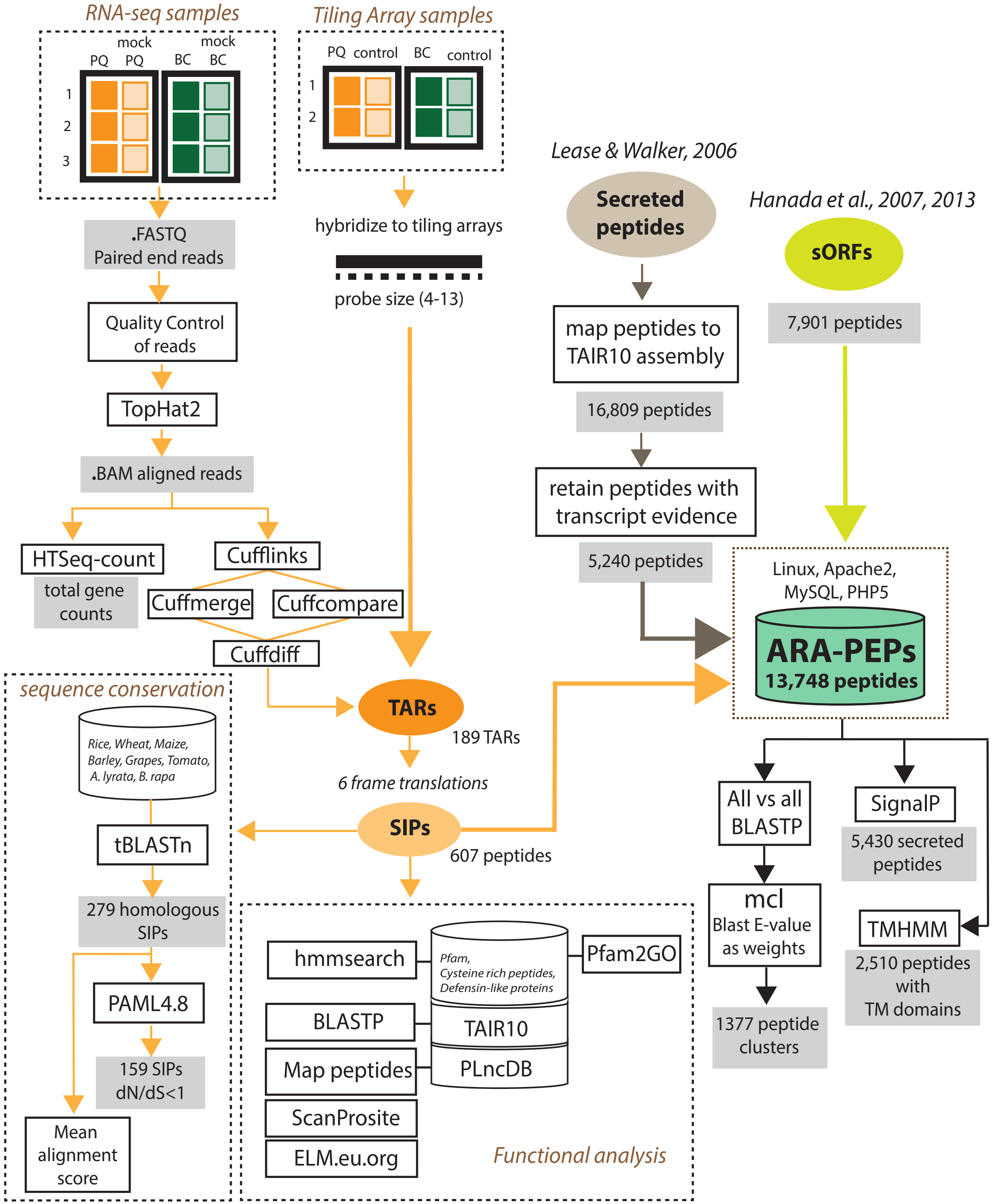

RNA-seq samples
PQ
mock PQ
BC
mock BC
1
2
3
Tiling Array samples
PQ control BC control
1
2
.FASTQ
Paired end reads
Quality Control of reads
TopHat2
.BAM aligned reads
HTSeq-count
total gene counts
Cufflinks
Cuffmerge
Cuffcompare
Cuffdiff
hybridize to tiling arrays
probe size (4-13)
Lease & Walker, 2006
Secreted peptides
map peptides to TAIR10 assembly
16,809 peptides
retain peptides with transcript evidence
5,240 peptides
Hanada et al., 2007, 2013
sORFs
7,901 peptides
Linux, Apache2, MySQL, PHP5
ARA-PEPs
13,748 peptides
TARs
189 TARs
6 frame translations
SIPs
607 peptides
sequence conservation
Rice, Wheat, Maize, Barley, Grapes, Tomato, A. lyrata, B. rapa
tBLASTn
279 homologous SIPs
PAML4.8
159 SIPs dN/dS<1
Mean alignment score
hmmsearch
Pfam, Cysteine rich peptides, Defensin-like proteins
Pfam2GO
BLASTP
TAIR10
Map peptides
PLncDB
ScanProsite
ELM.eu.org
Functional analysis
All vs all BLASTP
mcl
Blast E-value as weights
1377 peptide clusters
SignalP
5,430 secreted peptides
TMHMM
2,510 peptides with TM domains
